# Restoring Wnt6 signaling ameliorates behavioral deficits in MeCP2 T158A mouse model of Rett syndrome

**DOI:** 10.1038/s41598-020-57745-w

**Published:** 2020-01-23

**Authors:** Wei-Lun Hsu, Yun-Li Ma, Yen-Chen Liu, Derek J. C. Tai, Eminy H. Y. Lee

**Affiliations:** 1 Institute of Biomedical Sciences, Academia Sinica, Taipei, Taiwan; 2 Graduate Institute of Life Sciences, National Defense Medical Center, Taipei, Taiwan; 3 Molecular Neurogenetics Unit, Center for Genomic Medicine, Massachusetts General Hospital, Boston, Massachusetts USA; 4 Department of Neurology, Harvard Medical School, Boston, Massachusetts USA

**Keywords:** Autism spectrum disorders, Neurological disorders

## Abstract

The methyl-CpG-binding protein 2 gene, *MECP2*, is an X chromosome-linked gene encoding the MeCP2 protein, and mutations of *MECP2* cause Rett syndrome (RTT). Previous study has shown that re-expression of SUMO-modified MeCP2 in *Mecp2*-null neurons rescues synaptic and behavioral deficits in *Mecp2* conditional knockout mice, whereas about 12-fold decrease in *Wnt6* mRNA level was found in MeCP2K412R sumo-mutant mice. Here, we examined the role of Wnt6 in MeCP2 T158A mouse model of RTT. Results show that lentiviral delivery of Wnt6 to the amygdala ameliorates locomotor impairment and social behavioral deficits in these animals. MeCP2 T158A mice show decreased level of GSK-3β phosphorylation and increased level of β-catenin phosphorylation. They also show reduced level of MeCP2 SUMOylation. These alterations were also restored by lenti-Wnt6 transduction. Further, both BDNF and IGF-1 expressions are decreased in MeCP2 T158A mice. Overexpression of Wnt6 increases *Bdnf* and *Igf-1* promoter activity in HEK293T cells in a dose-dependent manner. Lenti-Wnt6 transduction to the amygdala similarly increases the mRNA level and protein expression of BDNF and IGF-1 in MeCP2 T158A mice. Moreover, environmental enrichment (EE) similarly ameliorates the locomotor and social behavioral deficits in MeCP2 T158A mice. One of the mechanisms underlying EE is mediated through enhanced MeCP2 SUMOylation and increased Wnt6 expression in these animals by EE.

## Introduction

Methyl-CpG-binding protein 2 (*MECP2*) is an X chromosome-linked gene that encodes the MeCP2 protein. MeCP2 contains two major domains, the methyl-DNA binding domain and the transcriptional repression domain, which are both structurally conserved^1^. MeCP2 was first found to function as a transcriptional repressor. It binds to the CpG island of methylated DNA and recruits co-repressors, such as the histone deacetylase (HDAC) complex, for transcriptional repression^2^. But a later study has indicated that MeCP2 could also function as a transcriptional activator^3^.

MeCP2 plays an important role in a few neuro-developmental disorders, and one of them is the Rett syndrome (RTT)^1,4,5^. RTT is a rare neurological and developmental disorder and it shares some common features with autism spectrum disorder^6^. The RTT patients usually develop normally at their infant stage, but regression develops afterwards. The abnormal behaviors usually include motor function deficits, cognitive impairment and other symptoms associated with mental retardation^1,7^. RTT is caused by mutations of the *MECP2* gene. So far, more than 200 mutations have been identified that are associated with RTT^8^. Some of these mutations are rare, but there are several most commonly seen mutations including T158M, R168X (X represents a premature stop codon), R133C, R106W, P152A, R306C and P376R, and the first three mutations account for approximately 32% of total RTT patients^9,10^. Our previous study reveals that six out of these seven *MECP2* mutations, except MECP2R168X as it lacks the C-terminal domain, show significantly decreased MeCP2 SUMOylation at Lys-412 compared with wild-type (WT) MECP2^11^. Moreover, re-expression of WT or the sumoylated form of MeCP2 in *Mecp2* conditional knockout mice rescues behavioral and synaptic deficits in these animals. Further cDNA microarray analyses indicate that the mRNA level of *Wnt6* is decreased for approximately 12-fold in the MeCP2K412R sumo-mutant group of animals compared with MeCP2 WT group of animals^11^. Wnt proteins and the Wnt/β-catenin pathway play a critical role during development^12^ and in adult brain function^13^. Also, it has been highlighted to be a signaling hub for autism^14^. Because all the *MECP2* mutations associated with RTT show decreased MeCP2 SUMOylation level and because *Wnt6* mRNA level is dramatically reduced in MeCP2K412R sumo-mutant animals^11^, these observations suggest that deficiency in Wnt6 signaling and its downstream effectors may play a role in the pathogenesis of RTT. To test this hypothesis, we examined whether restoration of Wnt6 signaling rescues the behavioral deficits in *Mecp2* mutant mice. We also studied the molecular mechanism of the rescuing effect of Wnt6 and how Wnt6 expression is regulated in the context of RTT. For this purpose, we have adopted the MeCP2 T158A mutant mice as an animal model of RTT^15^.

## Results

### Overexpression of Wnt6 in the amygdala reduces locomotor impairment and social behavior deficits in MeCP2 T158A mutant mice

**Figure 1:**
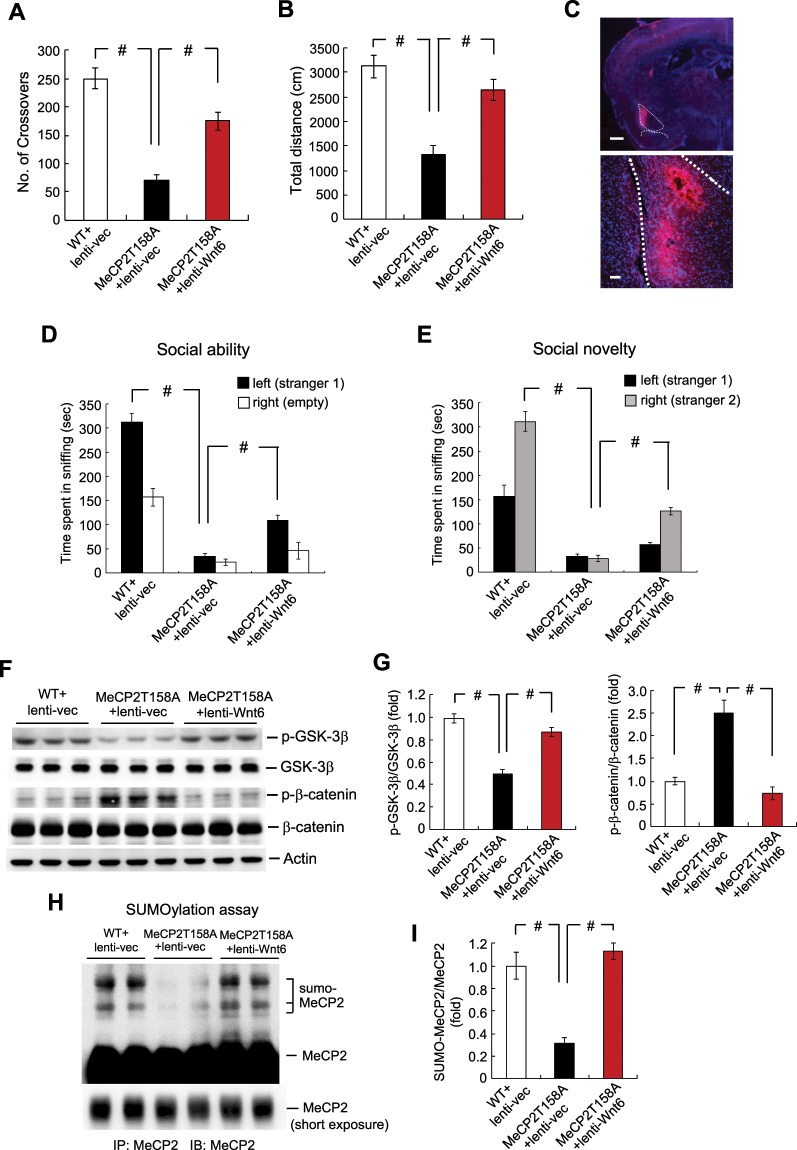
Overexpression of Wnt6 to the amygdala reduces locomotor impairment and social behavior deficits in MeCP2 T158A mutant mice. Animals were divided to three groups: WT mice received lenti-vector transduction, MeCP2 T158A mice received lenti-vector transduction and MeCP2 T158A mice received lenti-Wnt6 transduction to the amydgala. They were subjected to locomotor activity measure for 20 min 12 days later (n = 8 each group) and the **(A)** number of crossovers (F_2,21_ = 36.89, *P* < 0.001; q = 12.1, *P* < 0.001 comparing the WT + lenti-vector group with MeCP2 T158A + lenti-vector group and q = 7.03, *P* < 0.001 comparing the MeCP2 T158A + lenti-vector group with MeCP2 T158A + lenti-Wnt6 group) and **(B)** total distance travelled (F_2,21_ = 19.77, *P* < 0.001; q = 8.58, *P* < 0.001 comparing the WT + lenti-vector group with MeCP2 T158A + lenti-vector group and q = 6.31, *P* < 0.001 comparing the MeCP2 T158A + lenti-vector group with MeCP2 T158A + lenti-Wnt6 group) are shown. **(C)** Immunohistochemistry showing the location of lenti-mRFP-Wnt6 transduction and expression in the mouse amygdala. Scale bar equals 500 μm for the left panel and scale bar equals 100 μm for the right panel. The same animals were subjected to the **(D)** social ability test (for sniffing to stranger 1, F_2,19_ = 128.65, *P* < 0.001; q = 21.72, *P* < 0.001 comparing the WT + lenti-vector group with MeCP2 T158A + lenti-vector group and q = 5.59, *P* < 0.001 comparing the MeCP2 T158A + lenti-vector group with MeCP2 T158A + lenti-Wnt6 group) and **(E)** social novelty test (for sniffing to stranger 2, F_2,19_ = 107.48, *P* < 0.001; q = 20.28, *P* < 0.001 comparing the WT + lenti-vector group with MeCP2 T158A + lenti-vector group and q = 6.79, *P* < 0.001 comparing the MeCP2 T158A + lenti-vector group with MeCP2 T158A + lenti-Wnt6 group) one week later (n = 7 or 8 each group). **(F)** The amygdala tissue from these animals was dissected out and subjected to western blot analyses for the expression of phospho(p)Ser-9 GSK-3β, GSK-3β, phospho(p)Ser33/37/Thr41 β-catenin and β-catenin. A representative gel pattern is shown. **(G)** The quantified results are shown (n = 6 or 8 each group) (for p-GSK-3β over GSK-3β, F_2,17_ = 45.07, *P* < 0.001; q = 13.18, *P < *0.001 comparing the WT + lenti-vector group with MeCP2 T158A + lenti-vector group and q = 9.23, *P* < 0.001 comparing the MeCP2 T158A + lenti-vector group with MeCP2 T158A + lenti-Wnt6 group) (for p-β-catenin over β-catenin, F_2,17_ = 27.4, *P* < 0.001; q = 8.69, *P < *0.001 comparing the WT + lenti-vector group with MeCP2 T158A + lenti-vector group and q = 9.58, *P* < 0.001 comparing the MeCP2 T158A + lenti-vector group with MeCP2 T158A + lenti-Wnt6 group). **(H)** The same amygdala tissue lysates used above were also subjected to MeCP2 SUMOylation determination. A representative gel pattern is shown. **(I)** The quantified results are shown (n = 4 each group) (F_2,9_ = 26.25, *P* < 0.001; q = 8.05, *P < *0.001 comparing the WT + lenti-vector group with MeCP2 T158A + lenti-vector group and q = 9.52, *P* < 0.001 comparing the MeCP2 T158A + lenti-vector group with MeCP2 T158A + lenti-Wnt6 group). Data are expressed as mean ± SEM. ^#^*P* < 0.001.

Wnt signaling is mediated by the canonical pathway involving GSK-3β and β-catenin and the non-canonical pathway which is β-catenin-independent in general^16,17^. In this experiment, we examined the Wnt/β-catenin signaling pathway because this pathway was shown to regulate gene transcription, synaptic plasticity, neuronal function and mental disorders^18,19^. Animals were divided to the following three groups: WT mice received lenti-vector transduction, MeCP2 T158A mice received lenti-vector transduction and MeCP2 T158A mice received lenti-Wnt6 transduction. Because locomotor deficit is often seen in animal models of RTT^15^, we first examined their locomotor activity 12 days after lentiviral transductions. Results revealed that the MeCP2 T158A mutant mice showed significantly decreased number of crossovers in the activity chamber compared with WT mice, but this impairment was significantly rescued by lenti-Wnt6 transduction (Fig. 1A). The MeCP2 T158A mice also made less travelling in the activity chamber, but lenti-Wnt6 transduction similarly rescued this motor impairment (Fig. 1B). Immunohistochemical result showed the location of lenti-Wnt6 transduction and expression in the amygdala (Fig. 1C). Social behavior deficit is another symptom seen in RTT patients^1^ and in mouse models of RTT^15,20^. We also examined whether the MeCP2 T158A mutant mice exhibit social behavior deficits and whether these deficits could be rescued by Wnt6 overexpression. The same animals were subjected to social interaction measures one week after locomotor activity test. Results revealed that the WT mice spent more time sniffing to stranger 1 compared to the empty compartment during the social ability test, but this measure was dramatically decreased in MeCP2 T158A mice. However, this behavioral deficit was significantly, although partially, rescued in MeCP2 T158A mice receiving lenti-Wnt6 transduction (Fig. 1D). Similar results were obtained for the social novelty test. The WT mice spent more time sniffing to stranger 2 compared to stranger 1. This measure was dramatically decreased in MeCP2 T158A mice, but it was partially, yet significantly, rescued by lenti-Wnt6 transduction to MeCP2 T158A mice (Fig. 1E).

Animals were sacrificed after the social novelty test and their amygdala tissue was dissected out for various western blot analyses. Results indicated that the phosphorylation level of Ser-9 GSK-3β was significantly decreased in MeCP2 T158A mice compared with WT mice, but this phenomenon was restored by lenti-Wnt6 transduction to MeCP2 T158A mice (Fig. 1F). On the other hand, the phosphorylation level of β-catenin at Ser33/37/Thr41 was significantly increased in MeCP2 T158A mice compared with WT mice, but lenti-Wnt6 transduction similarly reversed β-catenin phosphorylation to the control level (Fig. 1F). The GSK-3β and β-catenin expression level remains similar among these three groups of animals. The quantified results are shown in Fig. 1G. Because transduction of lenti-Wnt6 successfully rescued the deficiency of GSK-3β phosphorylation and the increased level of β-catenin phosphorylation in MeCP2 T158A mice and these mice also have a reduced level of MeCP2 SUMOylation^11^, we next examined whether lenti-Wnt6 transduction also rescues the deficiency of MeCP2 SUMOylation in these animals. Results revealed that there is a consistent and dramatic decrease in the level of MeCP2 SUMOylation in MeCP2 T158A mice, but lenti-Wnt6 transduction successfully rescued this deficit (Fig. 1H). The quantified results are shown in Fig. 1I.

### Overexpression of Wnt6 increases the promoter activity and expression level of BDNF and IGF-1

**Figure 2:**
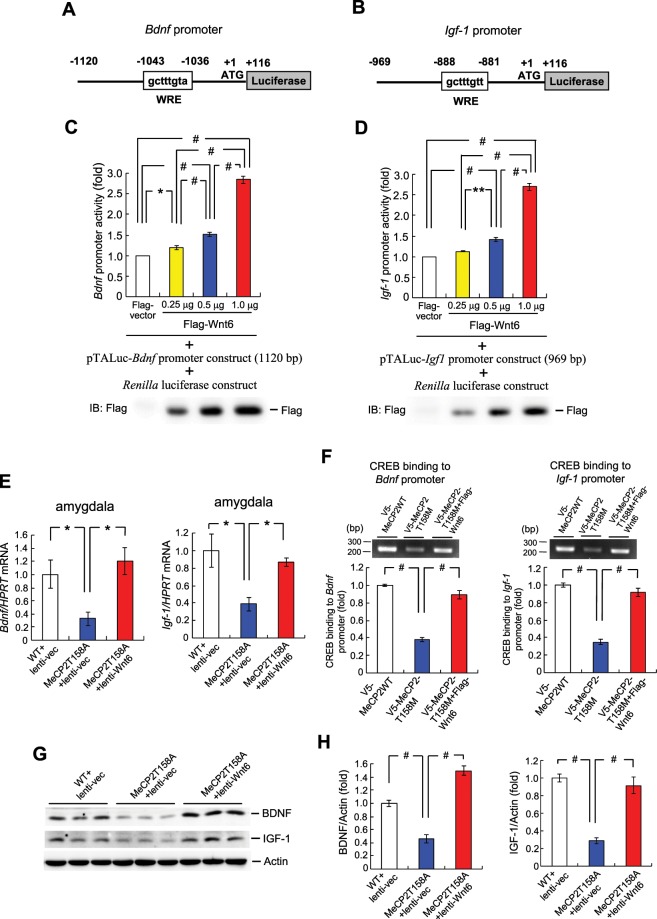
Overexpression of Wnt6 increases the promoter activity and expression level of BDNF and IGF-1. **(A)** *Bdnf* promoter-luciferase construct containing the WRE element is shown. **(B)** *Igf-1* promoter-luciferase construct containing the WRE element is shown. **(C)** Different amount of Flag-Wnt6 plasmid together with the pTALuc-*Bdnf* promoter construct (0.55 μg) and *Renilla* luciferase construct (0.05 μg) were transfected to HEK293T cells and *Bdnf* promoter activity was measured by luciferase assay (F_3,16_ = 257.03, *P* < 0.001). **(D)** Different amount of Flag-Wnt6 plasmid together with the pTALuc-*Igf-1* promoter construct (0.55 μg) and *Renilla* luciferase construct (0.05 μg) were transfected to HEK293T cells and *Igf-1* promoter activity was measured by luciferase assay (F_3,16_ = 183.53, *P* < 0.001). Western blot using anti-Flag antibody was adopted to confirm the transfection and expression of Flag-Wnt6 plasmids. Results are from five independent experiments. Lenti-vector was transducted to the amygdala of WT mice or MeCP2 T158A mice and lenti-Wnt6 was transducted to the amygdala of MeCP2 T158A mice. The amygdala tissue was dissected out and subjected to q-PCR analysis of **(E)** *Bdnf* mRNA level (F_2,17_ = 5.22, *P* < 0.05; q = 3.56, *P* < 0.05 comparing the WT + lenti-vector group with MeCP2 T158A + lenti-vector group and q = 4.32, *P* < 0.05 comparing the MeCP2 T158A + lenti-vector group with MeCP2 T158A + lenti-Wnt6 group) and *Igf-1* mRNA level (F_2,17_ = 5.35, *P* < 0.05; q = 4.5, *P* < 0.05 comparing the WT + lenti-vector group with MeCP2 T158A + lenti-vector group and q = 3.31, *P* < 0.05 comparing the MeCP2 T158A + lenti-vector group with MeCP2 T158A + lenti-Wnt6 group). **(F)** *V5-MeCP2WT*, *V5-MeCP2T158M* or *V5-MeCP2T158M* + Flag-Wnt6 plasmids were transfected to the mouse amygdala and ChIP assay for CREB binding to the *Bdnf* and *Igf-1* promoters are shown (n = 4 each group) (for CREB binding to the *Bdnf* promoter, F_2,9_ = 108.95, *P* < 0.001; q = 19.49, *P* < 0.001 comparing the WT + lenti-vector group with MeCP2 T158A + lenti-vector group and q = 16.21, *P* < 0.001 comparing the MeCP2 T158A + lenti-vector group with MeCP2 T158A + lenti-Wnt6 group) (for CREB binding to the *Igf-1* promoter, F_2,9_ = 87.13, *P* < 0.001; q = 17.17, *P* < 0.001 comparing the WT + lenti-vector group with MeCP2 T158A + lenti-vector group and q = 14.92, *P* < 0.001 comparing the MeCP2 T158A + lenti-vector group with MeCP2 T158A + lenti-Wnt6 group). **(G)** The amygdala tissue from the same animals was also subjected to western blot determination of BDNF and IGF-1 protein expression. A representative gel pattern is shown. **(H)** The quantified results are shown (n = 6 or 8 each group) (for BDNF over actin, F_2,17_ = 73.66, *P* < 0.001; q = 9.56, *P < *0.001 comparing the WT + lenti-vector group with MeCP2 T158A + lenti-vector group and q = 17.16, *P* < 0.001 comparing the MeCP2 T158A + lenti-vector group with MeCP2 T158A + lenti-Wnt6 group) (for IGF-1 over actin, F_2,17_ = 38.76, *P* < 0.001; q = 11.78, *P < *0.001 comparing the WT + lenti-vector group with MeCP2 T158A + lenti-vector group and q = 9.71, *P* < 0.001 comparing the MeCP2 T158A + lenti-vector group with MeCP2 T158A + lenti-Wnt6 group). Data are expressed as mean ± SEM. *P < 0.05 and ^#^*P* ≤ 0.001.

Promoter sequence analyses reveal that Wnt response element (WRE) is located on the promoter region of *Bdnf* (nt. −1043 ~ −1036) (Fig. 2A) and *Igf-1* (nt. −888 ~ −881) (Fig. 2B). BDNF release was found impaired in hippocampal neurons of *Mecp2* mutant mice and BDNF is dysregulated in RTT patient and in animal model of RTT^21,22^. Further, administration of a TrkB ligand restores synaptic plasticity and memory in RTT mice^23^. Moreover, IGF-1 treatment was shown to improve the RTT syndrome in patient^24^. Because of the important roles of BDNF and IGF-1 in RTT, we first examined whether Wnt6 signaling regulates *Bdnf* and *Igf-1* promoter activity. Results revealed that, when co-transfected with the *Bdnf* promoter construct and *Renilla* luciferase construct to HEK293T cells, Flag-Wnt6 plasmid transfection increased *Bdnf* promoter activity in a dose-dependent manner (Fig. 2C). Flag-Wnt6 transfection and expression were confirmed by western blot using anti-Flag antibody (Fig. 2C, lower panel). Similarly, Flag-Wnt6 plasmid, when co-transfected with the *Igf-1* promoter construct and *Renilla* luciferase construct to HEK293T cells, dose-dependently increased *Igf-1* promoter activity (Fig. 2D). Flag-Wnt6 transfection and expression were also confirmed by western blot using anti-Flag antibody (Fig. 2D, lower panel).

Next, we examined whether overexpression of Wnt6 actually restores *Bdnf* and *Igf-1* mRNA level in MeCP2 T158A mice. Lenti-Wnt6 vector (or lentivector) was transducted to the amygdala of MeCP2 T158A mice. WT animals received lentivector transduction and served as the control group. Results revealed that *Bdnf* mRNA level was significantly decreased in the amygdala of MeCP2 T158A mice, but lenti-Wnt6 transduction to MeCP2 T158A mice restored the *Bdnf* mRNA level to that of WT animals (Fig. 2E, left panel). Similarly, *Igf-1* mRNA level was also reduced in the amygdala of MeCP2 T158A mice, but lenti-Wnt6 transduction to MeCP2 T158A mice restored *Igf-1* mRNA expression to 80% of the control level (Fig. 2E, right panel). We have previously shown that CREB released from the HDAC1 repressor complex regulates *Bdnf* gene expression^11^. Here, we further examined whether CREB is responsible for this effect. Because of the important role of IGF-1 in RTT, we also examined CREB regulation of *Igf-1* gene expression. *V5-MeCP2WT* plasmid or *V5-MeCP2T158M* mutant plasmid was transfected to the mouse amygdala and chromatin immunoprecipitation (ChIP) assay was carried out to study this issue. In another group, *Flag-Wnt6* plasmid was co-transfected with *V5-MeCP2T158M* plasmid to the mouse amygdala and we examined whether Wnt6 overexpression restores the deficit in CREB binding to the *Bdnf* and *Igf-1* promoters in *V5-MeCP2T158M*-transfected animals. Results revealed that CREB directly binds to both the *Bdnf* and *Igf-1* promoters when *V5-MeCP2WT* plasmid was transfected, but its binding intensity was significantly decreased by *V5-MeCP2T158M* transfection. However, overexpression of Wnt6 successfully rescued the CREB binding deficit (Fig. 2F). Next, we examined whether overexpression of Wnt6 increased BDNF and IGF-1 protein expression. Results revealed that both the BDNF and IGF-1 expression level was dramatically decreased in MeCP2 T158A mice compared to WT mice, but lenti-Wnt6 transduction restored the expression level of both BDNF and IGF-1 (Fig. 2G). The quantified results are shown in Fig. 2H.

### Environmental enrichment (EE) upregulates MeCP2 SUMOylation and Wnt6, BDNF as well as IGF-1 expression and alleviates behavioral deficits in MeCP2 T158A mice

**Figure 3:**
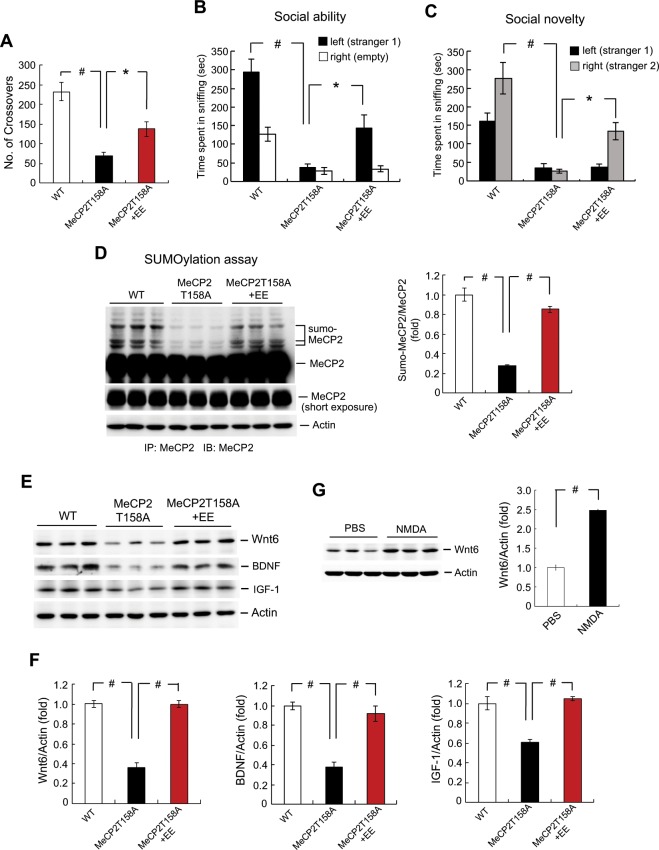
Environmental environment (EE) upregulates MeCP2 SUMOylation and Wnt6, BDNF as well as IGF-1 expression and alleviates behavioral deficits in MeCP2 T158A mice. **(A)** Animals were divided to three groups: WT mice housed in the home cage, MeCP2 T158A mice housed in the home cage and MeCP2 T158A mice subjected to EE. They were subjected to locomotor activity measure for 20 min at the end of EE (n = 8 each group) (F_2,21_ = 20.52, *P* < 0.001; q = 9.02, *P* < 0.001 comparing the WT group with MeCP2 T158A group and q = 3.81, *P* < 0.05 comparing the MeCP2 T158A group with MeCP2 T158A + EE group). The same animals were subjected to the **(B)** social ability test (for sniffing to stranger 1, F_2,19_ = 19.39, *P* < 0.001; q = 8.72, *P* < 0.001 comparing the WT group with MeCP2 T158A group and q = 3.47, *P* < 0.05 comparing the MeCP2 T158A group with MeCP2 T158A + EE group) and **(C)** social novelty test (for sniffing to stranger 2, F_2,19_ = 17.84, *P* < 0.001; q = 8.39, *P* < 0.001 comparing the WT group with MeCP2 T158A group and q = 3.5, *P* < 0.05 comparing the MeCP2 T158A group with MeCP2 T158A + EE group) one week later (n = 7 or 8 each group). **(D)** The amygdala tissue from these animals was dissected out and subjected to MeCP2 SUMOylation assay. The quantified result is shown at the lower panel (n = 6 or 8 each group) (F_2,17_ = 110.95, *P* < 0.001; q = 20.56, *P* < 0.001 comparing the WT group with MeCP2 T158A group and q = 14.87, *P* < 0.001 comparing the MeCP2 T158A group with MeCP2 T158A + EE group). **(E)** The same tissue lysate was also subjected to western blot determination of Wnt6, BDNF and IGF-1 expression. **(F)** The quantified results of Wnt6, BDNF and IGF-1 expression are shown (n = 6 or 8 each group) (for Wnt6, F_2,17_ = 87.93, *P* < 0.001; q = 15.79, *P* < 0.001 comparing the WT group with MeCP2 T158A group and q = 16.99, *P* < 0.001 comparing the MeCP2 T158A group with MeCP2 T158A + EE group) (for BDNF, F_2,17_ = 36.94, *P* < 0.001; q = 11.52, *P* < 0.001 comparing the WT group with MeCP2 T158A group and q = 9.44, *P* < 0.001 comparing the MeCP2 T158A group with MeCP2 T158A + EE group) (for IGF-1, F_2,17_ = 21.17, *P* < 0.001; q = 7.87, *P* < 0.001 comparing the WT group with MeCP2 T158A group and q = 8.24, *P* < 0.001 comparing the MeCP2 T158A group with MeCP2 T158A + EE group). **(G)** Mice received PBS or NMDA (6 mM) injection to their amygdala and Wnt6 expression was determined by western blot 1 h later (n = 6 each group) (t_1,10_ = 20.52, *P* < 0.001). Data are expressed as mean ± SEM. *P < 0.05 and ^#^*P* < 0.001.

Animal behavior is regulated by both genetic and environmental factors. Previous study has shown that EE at pre-weaning period improves motor coordination and motor learning in *Mecp2*^−/y^ mutant mice, and these changes are associated with restored long-term potentiation and increased BDNF level^25^. Further, EE decreases the motor coordination deficit on accelerating rotarod performance in *Mecp2*^+/−^ mutant mice^26^. But the molecular mechanism underlying the beneficial effect of EE to *Mecp2* mutant mice is less understood. Because we have presently found that enhanced Wnt6 signaling ameliorated the behavioral deficits and restored BDNF expression in MeCP2 T158A mice and we have earlier found that Wnt6 is regulated by MeCP2 SUMOylation^11^, here we examined whether MeCP2 SUMOylation and Wnt6 expression are regulated by EE. The MeCP2 T158A mice were either housed in their home cage or subjected to EE (1 h per day with 30 min in the morning and 30 min in the afternoon for 14 days consecutively). The WT mice housed in the home cage served as the control group. At the end of EE, they were subjected to locomotor activity measure and social interaction measure. Results revealed that MeCP2 T158A mice showed significant impairment in their locomotor activity, but EE significantly restored this impairment (Fig. 3A). The same animals were subjected to social interaction measure one week later. Results revealed that WT mice spent more time sniffing to stranger 1 than to the empty compartment for the social ability test. This measure was greatly reduced in MeCP2 T158A mice, but EE significantly, although partially, restored this behavioral deficit in MeCP2 T158A mice (Fig. 3B). Similar results were obtained with social novelty test. The WT mice spent more time sniffing to stranger 2 than to stranger 1. This measure was dramatically decreased in MeCP2 T158A mice, but EE similarly and partially rescued this behavioral deficit (Fig. 3C).

Animals were sacrificed after social behavior measures and their amygdala tissue was dissected out for determination of MeCP2 SUMOylation and Wnt6 expression. Results revealed that MeCP2 SUMOylation level was dramatically decreased in MeCP2 T158A mice, but this deficiency was significantly rescued by EE treatment (Fig. 3D). Similarly, Wnt6 expression was markedly decreased in MeCP2 T158A mice, but this reduction was completely restored by EE treatment (Fig. 3E). Because overexpression of Wnt6 restored BDNF and IGF-1 expression in MeCP2 T158A mice and EE restored Wnt6 level in MeCP2 T158A mice, it is expected that EE should also restore BDNF and IGF-1 level in MeCP2 T158A mice. This speculation was examined here. Results revealed that the expression level of BDNF and IGF-1 was consistently decreased in MeCP2 T158A mice, but EE markedly restored both BDNF and IGF-1 expression in these animals (Fig. 3E). The quantified results are shown in Fig. 3F. Further, EE was suggested to increase neuronal plasticity, at least in part, through the mediation of NMDA receptors^27^. Because EE rescues Wnt6 expression in MeCP2 T158A mice, it is likely that NMDA receptor activation may also upregulate Wnt6 expression. We examined this hypothesis here. Naive mice received PBS or NMDA injection to their amygdala and Wnt6 expression was determined 1 h later. Result revealed that NMDA administration markedly increased Wnt6 expression (Fig. 3G).

## Discussion

Previous study has shown that decreased MeCP2 SUMOylation is found in several *MECP2* mutations associated with RTT and nearly 12-fold reduction of *Wnt6* mRNA level is observed in MeCP2 sumo-mutant animals compared with MeCP2 WT animals^11^. In addressing the role of Wnt6, we have presently found that restoration of Wnt6/β-catenin signaling partially, but significantly, rescued locomotor activity impairment and social behavioral deficits in MeCP2 T158A mice. One of the underlying mechanisms is due to increased expression of BDNF and IGF-1 by Wnt6 overexpression. These results are consistent with the observations that activation of TrkB receptor restores memory in RTT mice^23^ and that recombinant human IGF-1 treatment recovers behavioral impairment in *Mecp2*^−/y^ mice^28^. They are also congruent with the literature that deregulation of Wnt signaling is associated with some mental and mood disorders^19^. It would be ideal to also measure the nuclear β-catenin level in MeCP2 T158A mice and in MeCP2 T158A mice transducted with lenti-Wnt6; however, the amygdala tissue lysate obtained from each animal is not enough to do co-immunoprecipitation for nuclear β-catenin, various western blotting and MeCP2 SUMOylation shown in Fig. 1F. Yet, the phosphorylation level of β-catenin is more important than the nuclear level of β-catenin in terms of Wnt6/β-catenin signaling. In addition, previous report shows that MeCP2 SUMOylation regulates Wnt6 expression and that BDNF and IGF-1 treatments both enhance MeCP2 SUMOylation. We have presently found that overexpression of Wnt6 rescued downregulation of BDNF and IGF-1 in MeCP2 T158A mice. These results implicate that Wnt6 may upregulate MeCP2 SUMOylation through increased BDNF and IGF-1 expression. These results together also suggest that there may exist a positive loop of regulation between MeCP2 SUMOylation and Wnt6 expression that is beneficial to RTT. This explanation is supported by our observation that overexpression of Wnt6 rescued MeCP2 SUMOylation deficit in MeCP2 T158A mice. But the molecular mechanism underlying this regulation requires further investigation. Further, the present result from ChIP assay suggests that BDNF and IGF-1 expression is regulated by CREB directly, and the *MeCP2T158M* mutant, which manifests a significant decrease in MeCP2 SUMOylation level^11^, also showed significant reduction in CREB binding to the *Bdnf* and *Igf-1* promoters. These deficits could be rescued by Wnt6 overexpression. On the other hand, although overexpression of Wnt6 effectively ameliorated these behavioral deficits, it did not completely rescue these deficits. One possible explanation is that genes other than *Wnt6* also play important roles in alleviating RTT. This explanation is supported by our earlier finding that many genes show several-fold downregulation in their mRNA levels in MeCP2 sumo-mutant animals^11^. Besides, complicated molecular mechanisms underlie the pathology of RTT, and MeCP2 T158M mutation is the most severe mutation in RTT patients^28^, it is conceivable that restoration of Wnt6/β-catenin signaling is not sufficient to completely rescue these behavioral deficits. Moreover, lenti-Wnt6 vector transduction was made only to a limited area in the amygdala. Neuronal activation in this area may not be sufficient for full behavioral recovery.

Previous studies have shown that EE at pre-weaning period improves motor function in *Mecp2*^−/y^ mice^25^ and EE ameliorates motor coordination deficit in *Mecp2*^+/−^ mutant mice^26^. In the present study, we have found that EE significantly improved locomotor activity in MeCP2 T158A mice. Extended from this result, we have found that EE also improved the social interaction behavior in MeCP2 T158A mice. In examination of the underlying mechanism, we found that EE enhanced the level of MeCP2 SUMOylation and the expression level of Wnt6, BDNF and IGF-1 in the amygdala of these animals. These results are consistent with our earlier finding that *Wnt6* mRNA expression is strongly regulated by MeCP2 SUMOylation and enhanced MeCP2 SUMOylation rescues the behavioral deficits in *Mecp2* conditional knockout mice^11^. Thus, we have provided novel molecular mechanisms of EE in the context of RTT. These results are also congruent with another report showing that EE improves memory function in vascular dementia rats associated with activation of Wnt3a signaling^29^. It is conceivable that EE activates many more genes other than *Wnt6*, yet EE could not completely rescue the behavioral deficits of MeCP2 T158A mice. These results suggest that certain defects in MeCP2 T158A mice are not able to be modulated by enhanced neuronal plasticity.

In this study, we found that EE and NMDA administration both increased the expression of Wnt6. We have earlier found that NMDA treatment enhances MeCP2 SUMOylation^11^. Because NMDA receptor activation mediates, in part, the effect of EE^27^, these results together suggest that EE may increase MeCP2 SUMOylation and upregulate Wnt6 expression through the mediation of NMDA receptors. Moreover, *Mecp2T158M* mutation shows decreased methyl-DNA binding^11^, but we have presently found that EE enhanced Wnt6 expression in MeCP2 T158A mice. Whether EE alters MeCP2 methyl-DNA binding of these mice or that EE increases Wnt6 expression in these animals through other mechanisms, such as decreased association between MeCP2T158A and HDAC1, requires further investigation.

In the present study, the MeCP2 T158A mice showed both locomotor activity impairment and social behavior deficits. The result of locomotor activity impairment is consistent with the finding that locomotor deficit is often seen in animal models of RTT^15^. It is possible that the observed social behavior deficits are partly contributed by the motor function impairment in these animals. However, the extent of social behavior impairment is greater than that of motor activity impairment comparing the MeCP2 T158A mice to WT mice (approximately 90% vs. 70%). This result suggests that *Mecp2T158A* mutation indeed affects social behavior *per se*. However, the amygdala is not primarily involved in motor control and there is little literature linking dysfunction of emotion control regulated by the amygdala and motor behavior, yet we have found that overexpression of Wnt6 in the amygdala partially rescued locomotor activity impairment in MeCP2 T158A mice. Because the amygdala is known to regulate dopamine release from the nucleus accumbens, an area regulates motor behavior^30^, it is possible that overexpressed Wnt6 regulates the projection from the amygdala to the nucleus accumbens and dopamine release from this area, that consequently regulates locomotor activity. But it is possible that other amygdala pathways are also involved in motor regulation. For example, anatomical tracing study reveals a major projection from the basolateral amygdala to the rostral cingulate motor cortex and a light projection from the basolateral amygdala to the caudal cingulate motor cortex in the rhesus monkey^31^. These projections may regulate certain emotion-related motor functions. To further elaborate the underlying mechanism, it would be helpful to directly transduct the Wnt6 vector to the nucleus accumbens or the motor cortex of MeCP2 T158A mice and examine their motor behaviors.

**Figure 4:**
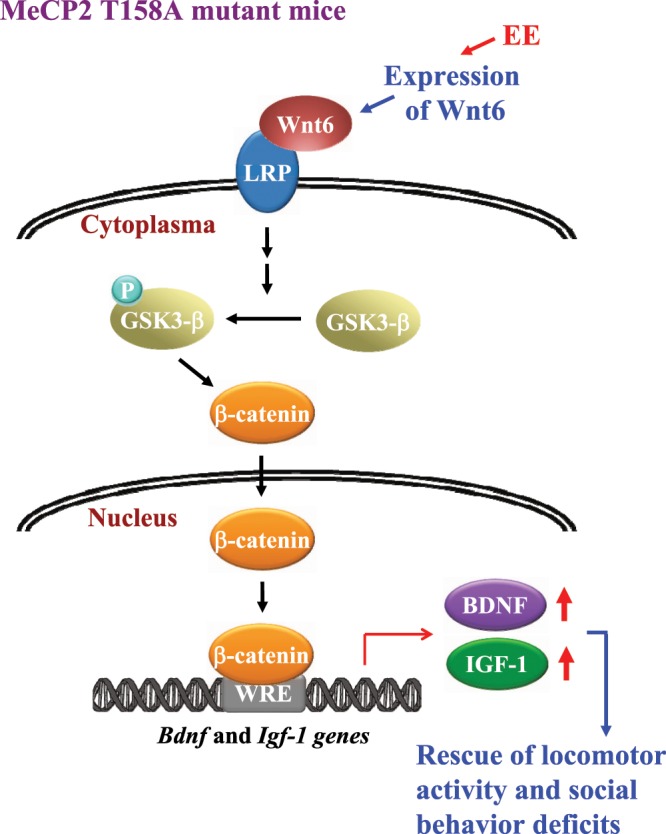
Schematic diagram showing that Wnt6 signaling is impaired in MeCP2 T158A mice and Wnt6 expression rescues the locomotor activity impairment and social behavior deficits in MeCP2 T158A mice. Further, Wnt6 expression is regulated by environmental enrichment. EE: environmental enrichment.

Wnt proteins play important roles in development, synapse formation, neuronal circuitry, cognitive function and certain neurological disorders. Here we first demonstrate the deficiency of Wnt6/β-catenin signaling in MeCP2 T158A mutant mice and restoring Wnt6/β-catenin signaling rescues various behavioral deficits in this mouse model of RTT (Fig. 4). These results shed light on the molecular mechanism underlying RTT and may lead to novel therapeutic options for RTT patients.

## Materials and Methods

### Animals

Adult male (2–3 months) *Mecp2*^*T158A/y*^ mice were used in this study. They were purchased from Jackson Laboratory (Bar Harbor, ME, USA; strain name: B6N.129(Cg)-Mecp2tm1.1Joez/J, stock number: 017741) and were bred at the Animal Facility of the Institute of Biomedical Sciences (IBMS), Academia Sinica in Taiwan. All the animals were housed and maintained on a 12/12 h light/dark cycle (light on at 6:30 am) with food and water continuously available. Experimental procedures followed the guidelines and ethical regulations of Animal Use and Care of the National Institute of Health and were approved by the Animal Committee of IBMS, Academia Sinica.

### Lentiviral vector construction and preparation

For construction of Flag-Wnt6 lentivitral vector, full-length Flag-Wnt6 fusion plasmid was sub-cloned into the lentiviral vector pLenti-Tri-cistronic (ABM, Richmond, BC, Canada) by amplifying the construct with the following primers: 5′-ATCGAGTACTGCCACCATGGATTACAAGGATGACGACGATAAG-3′ (forward) and 5′-ATCGGGTACCTCAGAGGCACAGGCTGAGTTC-3′ (reverse). The PCR product was sub-cloned between *ScaI* and *KpnI* sites of the lentiviral vector. For lentivirus packaging, HEK293LTV cells (Cell Biolabs, San Diego, CA) were transfected with 1.5 µg of psPAX2 (Addgene plasmid #12260), 0.5 µg of pMD2.G (Addgene plasmid #12259), and 2 µg of Flag-Wnt6WT or 2 µg of pLenti-Tri-cistronic as the control using 10 µl of Lipofectamine 2000 in 6-well cell culture dish. Lentiviral particles were collected using the speedy lentivirus purification solution (ABM) according to the manufacturer’s protocols. Cell culture medium containing lentiviral particles was harvested for two to three times at 12 h interval until 36 h after transfection, and it was kept at 4 °C for the collecting period. The collected culture medium was further clarified by centrifugation at 2,500 × g for 10 min and filtrated through a 0.45 µm syringe filter. The speedy lentivirus purification solution (ABM) was added into filtrated supernatant (1:9, v/v) containing lentiviral particles and mixed thoroughly by inversion. The lentiviral supernatant was centrifuged at 5,000 × g at 4 °C for 10 min. Supernatant was then discarded and the viral pellet was re-suspended in ice cold PBS. After titration, the viral stock was stored at −80 °C in aliquots. The lentivirus titer was determined by using the lentivirus qPCR Titer Kit (ABM) according to the manufacturer’s protocols (ABM). The titer of the pLenti-Wnt6WT vector was 1 × 10^8^ IU/ml.

### Locomotor activity measure

Locomotor activity was measured in the Digiscan Animal Activity Monitor System as described previously^32^. Briefly, the activity monitor is 16 inches in square with 16 × 16 horizontal by vertical infrared sensors and it is divided to nine sub-regions. These sensors were used to localize the animal’s floor position. The number of crossovers between sub-regions and the total distance travelled of each animal was recorded during the 20 min measurement period.

### Three-chamber social ability and social novelty measures

Social ability and social novelty measures were conducted in a three-chamber cage at the following specifications: 60 × 40 × 22 cm (L × W × H). The procedures used for these measures were adopted from that of a previous study^11^. The chamber is divided into three compartments with the left and right compartments of 21 cm in length and the middle compartment of 18 cm in length. There were two additional cylinder chambers with 15 cm in height and 10 cm in diameter placed in both the left and right compartments. During the social ability test, a stranger 1 mouse was placed inside the cylinder in the left compartment with the cylinder on the right compartment empty. The test subject was placed in the middle chamber for 10 min and its sniffing time toward stranger 1 and the empty chamber was recorded. At the end of the test, the test subject and stranger 1 were taken out and the chambers were cleaned. Ten minutes later, the test subject and stranger 1 were placed back to their original chambers, respectively. Meanwhile, a stranger 2 mouse was placed in the cylinder on the right compartment. The sniffing time of the test subject to stranger 1 and stranger 2 was recorded during the 10 min observation period and it is regarded as the social novelty measure.

### Statistical analysis

All the data are presented as mean values ± SEM. Data were analyzed by Student’s *t*-test or one-way analysis of variance (ANOVA) followed by Newman-Keuls multiple comparisons (represented by q value). Values of *P* < 0.05 were considered statistically significant (*P < 0.05, **P < 0.01 and ^#^*P* < 0.001).

## Supplementary information

Supplementary Methods.

## References

[1] Amir RE (1999). Rett syndrome is caused by mutations in X-linked MECP2, encoding methyl-CpG-binding protein 2. Nature genetics.

[2] Nan X (1998). Transcriptional repression by the methyl-CpG-binding protein MeCP2 involves a histone deacetylase complex. Nature.

[3] Chahrour M (2008). MeCP2, a key contributor to neurological diseases, activates and represses transcription. Science.

[4] Guy J, Cheval H, Selfridge J, Bird A (2011). The role of MeCP2 in the brain. Annual review of cell and developmental biology.

[5] Moretti P, Zoghbi HY (2006). MeCP2 dysfunction in Rett syndrome and related disorders. Current opinion in genetics & development.

[6] Percy AK (2011). Rett syndrome: exploring the autism link. Archives of neurology.

[7] Swanberg SE, Nagarajan RP, Peddada S, Yasui DH, LaSalle JM (2009). Reciprocal co-regulation of EGR2 and MECP2 is disrupted in Rett syndrome and autism. Human molecular genetics.

[8] Miltenberger-Miltenyi G, Laccone F (2003). Mutations and polymorphisms in the human methyl CpG-binding protein MECP2. Human mutation.

[9] Adegbola AA, Gonzales ML, Chess A, LaSalle JM, Cox GF (2009). A novel hypomorphic MECP2 point mutation is associated with a neuropsychiatric phenotype. Human genetics.

[10] Adkins NL, Georgel PT (2011). MeCP2: structure and function. Biochemistry and cell biology.

[11] Tai DJ (2016). MeCP2 SUMOylation rescues Mecp2-mutant-induced behavioural deficits in a mouse model of Rett syndrome. Nature communications.

[12] van Amerongen R, Nusse R (2009). Towards an integrated view of Wnt signaling in development. Development.

[13] Kwan V, Unda BK, Singh KK (2016). Wnt signaling networks in autism spectrum disorder and intellectual disability. Journal of neurodevelopmental disorders.

[14] Caracci MO, Avila ME, De Ferrari GV (2016). Synaptic Wnt/GSK3beta Signaling Hub in Autism. Neural plasticity.

[15] Goffin D (2011). Rett syndrome mutation MeCP2 T158A disrupts DNA binding, protein stability and ERP responses. Nature neuroscience.

[16] Davis EK, Zou Y, Ghosh A (2008). Wnts acting through canonical and noncanonical signaling pathways exert opposite effects on hippocampal synapse formation. Neural development.

[17] Salinas PC, Zou Y (2008). Wnt signaling in neural circuit assembly. Annual review of neuroscience.

[18] Fortress AM, Frick KM (2016). Hippocampal Wnt signaling: memory regulation and hormone interactions. The Neuroscientist: a review journal bringing neurobiology, neurology and psychiatry.

[19] Oliva CA, Vargas JY, Inestrosa NC (2013). Wnt signaling: role in LTP, neural networks and memory. Ageing research reviews.

[20] Moretti P, Bouwknecht JA, Teague R, Paylor R, Zoghbi HY (2005). Abnormalities of social interactions and home-cage behavior in a mouse model of Rett syndrome. Human molecular genetics.

[21] Li W, Calfa G, Larimore J, Pozzo-Miller L (2012). Activity-dependent BDNF release and TRPC signaling is impaired in hippocampal neurons of Mecp2 mutant mice. Proceedings of the National Academy of Sciences of the United States of America.

[22] Li W, Pozzo-Miller L (2014). BDNF deregulation in Rett syndrome. Neuropharmacology.

[23] Li W (2017). A small-molecule TrkB ligand restores hippocampal synaptic plasticity and object location memory in Rett syndrome mice. Disease models & mechanisms.

[24] Pini G (2014). Repeated insulin-like growth factor 1 treatment in a patient with rett syndrome: a single case study. Frontiers in pediatrics.

[25] Lonetti G (2010). Early environmental enrichment moderates the behavioral and synaptic phenotype of MeCP2 null mice. Biological psychiatry.

[26] Kondo M (2008). Environmental enrichment ameliorates a motor coordination deficit in a mouse model of Rett syndrome–Mecp2 gene dosage effects and BDNF expression. The European journal of neuroscience.

[27] Tang YP, Wang H, Feng R, Kyin M, Tsien JZ (2001). Differential effects of enrichment on learning and memory function in NR2B transgenic mice. Neuropharmacology.

[28] Castro J (2014). Functional recovery with recombinant human IGF-1 treatment in a mouse model of Rett Syndrome. Proceedings of the National Academy of Sciences of the United States of America.

[29] Jin X (2017). Environmental enrichment improves spatial learning and memory in vascular dementia rats with activation of Wnt/beta-catenin signal pathway. Medical science monitor: international medical journal of experimental and clinical research.

[30] Jackson ME, Moghaddam B (2001). Amygdala regulation of nucleus accumbens dopamine output is governed by the prefrontal cortex. The Journal of neuroscience.

[31] Morecraft RJ (2007). Amygdala interconnections with the cingulate motor cortex in the rhesus monkey. The Journal of comparative neurology.

[32] Wu HC, Chen KY, Lee WY, Lee EHY (1997). Antisense oligonucleotides to corticotropin-releasing factor impair memory retention and increase exploration in rats. Neuroscience.

