# Supplementary material for: Restoring Wnt6 signaling ameliorates behavioral deficits in MeCP2 T158A mouse model of Rett syndrome
Source: Sci Rep. 2020 Jan 23;10:1074. doi: 10.1038/s41598-020-57745-w (PMC6978308; doi:10.1038/s41598-020-57745-w)
Supplement: Supplementary file 1 — Supplementary Methods. [file 41598_2020_57745_MOESM1_ESM.pdf]

Restoring Wnt6 signaling ameliorates behavioral deficits

in MeCP2 T158A mouse model of Rett syndrome

Wei-Lun Hsu,[1] Yun-Li Ma,[1] Yen-Chen Liu,[2] Derek JC Tai[3,4] and Eminy HY Lee[1]*

[1]Institute of Biomedical Sciences, Academia Sinica, Taipei, Taiwan

[2]Graduate Institute of Life Sciences, National Defense Medical Center, Taipei, Taiwan

[3]Molecular Neurogenetics Unit, Center for Genomic Medicine, Massachusetts General Hospital, Boston, Massachusetts, USA

[4]Department of Neurology, Harvard Medical School, Boston, Massachusetts, USA

Supplementary methods

Hippocampal lysate and cell lysate preparation

Mouse hippocampal tissue and HEK293T cells were lysed by brief sonication in lysis buffer containing 50 mM Tris-HCl (pH 7.4), 150 mM NaCl, 2 mM EDTA and 1% IGEPAL CA-630. One tablet of protease inhibitor cocktail (cOmplete™ ULTRA Tablets, Mini, EDTA-free, EASYpack, Roche, Mannheim, Germany) and one tablet of phosphatase inhibitor (PhosSTOP, Roche) were added to each 10 ml of the lysis buffer before sonication. The homogenate was centrifuged at 14000 x rpm for 10 min at 4 °C. The supernatant was collected and stored at -80 °C until further analysis.

Plasmid construction and DNA transfection

For construction of the Flag-tagged Wnt6 plasmid, full-length Wnt6 was cloned by amplifying the mouse *Wnt6* cDNA (accession # NM_009526.3) with primers 5’-ATCGGAATTCATGCTGCCGCCGGTGCCCTCC-3’ (forward) and 5’-ATCGAAGCTTTCAGAGGCACAGGCTGAGTTC-3’ (reverse). The PCR product was sub-cloned between the *EcoRI* and *HindIII* sites of the mammalian expression vector pCMVTag2B. HEK293T cells were maintained in Dulbecco’s modified Eagle’s medium containing 10% fetal bovine serum and incubated at 37 °C in a humidified atmosphere with 5% CO_2. Transfection to HEK293T cells was made

using the Lipofectamine 2000 reagent (Invitrogen, Carlsbad, CA) in 12-well culture plates according to the manufacturer's instructions. For construction of the V5-tagged *MeCP2* plasmid, full-length *Mecp2* was cloned by amplifying the rat hippocampal *Mecp2* cDNA (accession # NM_022673) with primers 5'-ATTTGCGGCCGCCACCATGGTAGCTGGGATGTTAG-3' and 5'-TAACCGCGGGCTAACTCTCTCGGTCAC-3'. The PCR product was sub-cloned between the *NotI* and *SacII* sites of the mammalian expression vector pcDNA3.1-V5-His. *V5-MeCP2T158M* mutant plasmid was generated using the QuickChange Site-Directed Mutagenesis Kit (Stratagene, La Jolla, CA) as described previously[33]. Mice were anesthetized with pentobarbital (40 mg/kg, i.p.) and subjected to stereotaxic surgery. *V5-MeCP2WT* or *V5-MeCP2T158M* plasmid was directly injected to the mouse amygdala at a rate of 0.1 μl/min. A total of 0.25 μl was injected to each side of amygdala. Transient plasmid DNA transfection was conducted using the non-viral transfection agent polyethyleneimine (PEI) and we have previously demonstrated that PEI does not produce toxicity to neurons[34]. Briefly, plasmid DNA was diluted in 5% glucose to a stock concentration of 2.77 μg/μl. Branched PEI of 25 kDa (Sigma) was diluted to 0.1 mM concentration in 5% glucose and added to the DNA solution. Immediately before injection, 0.1 mM PEI was added to reach a ratio of PEI nitrogen per DNA phosphate equals to 10. The mixture was subjected to vortex

for 30 sec and allowed to equilibrate for 15 min.

Promoter luciferase assay

HEK293T cells were maintained in Dulbecco's modified Eagle's medium containing 10% fetal calf serum and incubated at 37 °C in a humidified atmosphere with 5% CO_2. For *Bdnf* and *Igf-1* reporter assay, the 1.12 kb length of the mouse TA-Luc *Bdnf*-luciferase reporter plasmid (0.55 μg) or the 0.97kb length of the mouse TA-Luc *Igf-1*-luciferase reporter plasmid (0.55 μg) and the *Renilla* luciferase-encoding internal control plasmid phRG-TK (0.05 μg) were co-transfected with the Flag-Wnt6 plasmid (or Flag-vector) (0.25, 0.5 and 1.0 μg) to HEK293T cells using Lipofectamine 2000 (4 μl). The primer set used to obtain the 1.12 kb length mouse *Bdnf* promoter is as follows: the forward primer: 5'-ATCGGGTACCGACTCTGTAGGAAAGTTC-3' (nucleotide -1117 to -1100) and the reverse primer: 5'-ATCGCTCGAGCATCGCTTCTGAAGTACA-3' (nucleotide +3 to -15). The primer set used to obtain the 0.96 kb length mouse *Igf-1* promoter is as follows: the forward primer: 5'-ATCGGGTACCGAGAACAATACAGCCAAC-3' (nucleotide -966 to -949) and the reverse primer: 5'-ATCGCTCGAGCATCGCTTCTGAAGTACA-3' (nucleotide +3 to -15). Luciferase activity assay was performed 48 h later using the Dual-Glo luciferase assay

system (Promega) and the TD-20/20 Luminometer (Turner Designs Hydrocarbon Instruments). The relative *Bdnf* and *Igf-1* luciferase activity was normalized to that of the *Renilla* luciferase activity.

Quantitative real-time PCR (Q-PCR)

Total RNA was isolated from 20 mg of hippocampal CA1 tissue using RNeasy Mini Kit (Qiagen, Germantown, MD) according to the manufacturer’s instructions. The RNA samples were re-suspended in nuclease-free water and quantified spectrophotometrically at 260 nm. All RNA samples had an *A260:A280* value between 1.8 and 2.0. cDNA synthesis was carried out by using the QuantiTect Reverse Transcription Kit (Qiagen) according to the manufacturer's protocols. The cDNA stock was stored at -20 °C. Quantitative PCR for *Bdnf* or *Igf-1* and the endogenous control gene *HPRT* was carried out using the iQ SYBR Green Supermix (Bio-rad). The primer sequences for *Bdnf* were: 5'-CTGCCTAGATCAAATGGAGCTTCT-3' (forward) and 5'-GGAAATTGCATGGCGGAGGTAA-3' (reverse). The primer sequences for *Igf-1* were: 5'-TCATGTCGTCTTCACACCTCTT-3' (forward) and 5'-CCACACACGAACTGAAGAGCAT-3' (reverse). The primer sequences for HPRT were: 5'-GCCGACCGGTTCTGTCAT-3' (forward) and

5'-TCATAACCTGGTTCATCATCACTAATC-3' (reverse). Amplification was performed using the Rotor-Gene Q Real Time PCR system (Qiagen), and the reaction condition followed the manufacturer’s protocols. The thermal cycler protocol used is as follows: 95 °C for 10 min, 95 °C for 10 s, and 60 °C for 30 s for 40 cycles. The cycle threshold (Ct) values and related data were analyzed using the Rotor-Gene Q Real Time PCR System Software (Qiagen). The expression level of *Bdnf* and *Igf-1* was normalized to that of *HPRT*. The relative expression level (in fold) was determined by using the $2^{-(\triangle\triangle Ct)}$ method.

Western blot

Cell lysates were resolved by 8~12% SDS-PAGE and transferred onto the PVDF membrane (Millipore, Bedford, MA). Western blot was conducted using the following antibodies: anti-Wnt6 (Abcam, Cambridge, UK), anti-GSK-3β (Cell Signaling, Danvers, MA), anti-pSer9-GSK-3β (Cell Signaling), anti-β-catenin (Cell Signaling), anti-pSer33/37/Thr41-β-catenin (Cell Signaling), anti-BDNF (Abcam), anti-IGF-1 (Santa Cruz Biotechnology, Dallas, TX), anti-MeCP2 (Cell Signaling), anti-Flag (Sigma-Aldrich) and anti-actin (Millipore). The secondary antibody used was HRP-conjugated goat-anti rabbit IgG antibody or goat-anti mouse IgG antibody (Jackson ImmunoResearch, West Grove, PA). Membrane was developed by reacting

with chemiluminescence HRP substrate (Millipore) and exposed to the LAS-3000 image system (Fujifilm, Tokyo, Japan) for visualization of protein bands. The protein bands were quantified by using the NIH Image J Software.

MeCP2 SUMOylation assay for the amygdala tissue

The amygdala tissue lysate was prepared in the same way as that prepared for western blot. For immunoprecipitation of MeCP2, the clarified lysate (0.5 mg) was immunoprecipitated with 3 μl of anti-MeCP2 antibody (Cell Signaling) at 41 °C overnight. The protein A agarose beads (30 ml, 50% slurry, GE Healthcare, Barrington, IL) were added to the IP reaction product to catch the immune complex at 4 °C for 3 h. The immune complex on beads were washed three times with washing buffer containing 20 mM HEPES (pH 7.4), 150 mM NaCl, 1 mM EDTA, 1% IGEPAL CA-630, 1 mM DTT, 50 mM β-glycerophosphate, 50 mM NaF, 10 mg/ml PMSF, 4 mg/ml aprotinin, 4 mg/ml leupeptin and 4 mg/ml pepstatin and subjected to *in vitro* SUMOylation reaction with the addition of recombinant PIAS1 protein (3 μl, Enzo Life Sciences, Farmingdale, NY), E1 (1 μl), E2 (1 μl) and the SUMO1 (0.5 μl) proteins provided in the kit. *In vitro* SUMOylation assay was performed according to that described previously[35] using the SUMO link kit according to the manufacturer’s instructions (Active Motif, Carlsbad, CA) and boiled in Laemmli sample buffer at 95

°C for 10 min. The *in vitro* SUMOylation product was subjected to 8% SDS-PAGE followed by transferring onto the PVDF membrane (Millipore). The membrane was immunoblotted with anti-MeCP2 antibody (Cell Signaling).

Chromatin immunoprecipitation (ChIP) assay

ChIP assay was performed according to that described previously[36] by using the ChIP assay kit from Millipore (Catalog No. 17-10085). Briefly, the mouse amygdala tissue was washed with 1 x ice-cold PBS and fixed with 1% formaldehyde by adding formaldehyde to the ice-cold PBS for 10 min. After adding glycine to quench the un-reacted formaldehyde, tissue was homogenized and re-suspended in cell lysis buffer plus protease inhibitor cocktail II, then changed to nuclear lysis buffer plus protease inhibitor cocktail II for sonication. The chromatin was immunoprecipitated using rabbit anti-CREB antibody (Cell Signaling, Danvers, MA). DNA purified from the immunoprecipitated samples was subjected to PCR reaction. The forward primer used for the *Bdnf* promoter is: 5'-TCATCACTCACGACCACG-3' (nucleotide -827 to -810) and the reverse primer is: 5'-CTTGTTGCTTTTCTGCAC-3' (nucleotide -623 to -606). The PCR product of the *Bdnf* promoter is 222 bps in length. The forward primer used for the *Igf-1* promoter is: 5'-GGAGATAGTCTCTTCTTC-3' (nucleotide -618 to -601) and the reverse primer is: 5'-TCTGACAGGGTTAGCAGA-3'

(nucleotide -416 to -399). The PCR product of the *Igf-1* promoter is 220 bps in length. The PCR product was separated by 2% agarose gel electrophoresis.

Intra-amygdala lentiviral vector injection

Mice were anesthetized with pentobarbital (40 mg/kg, intraperitoneally) and subjected to stereotaxic surgery without cannulation. Lentiviral vectors were directly injected to the lateral amygdala after animals recovered from the surgery. The coordinates for the lateral amygdala are: 0.5 mm posterior to the bregma, ±3.3 mm lateral to the midline and 4.8 mm ventral to the skull surface. A volume of 0.25 µl was injected to each side of amygdala. The infusion rate was 0.1 µl/min. Locomotor activity was measured 12 days after lentiviral vector transduction. Social behavior was measured 7 days after locomotor activity measure. Mice were sacrificed after the social behavioral test. Their brains were removed and the amygdala tissue was punched out for further analyses.

Immunohistochemistry

For visualization of lentiviral Wn6 transduction and expression in the amygdala, lenti-mRFP-Wnt6 vector was constructed and transducted to the amygdala in separate mice. The procedures used were adopted from that of a previous study[11]. Briefly,

brain sections were rinsed with 1 X PBS for 10 min at room temperature and permeabilized with pre-cold EtOH/CH_3COOH (95%:5%) for 10 min, followed by 1 X PBS for 10 min three times. The sections were pre-incubated in a blocking solution containing 3% normal goat serum, 3% BSA, and 0.2% Triton X-100 in 1 X PBS for 2 h at room temperature, followed by 1 X PBS for 10 min for three times.

Photomicrographs were taken using a Zeiss LSM700 Stage confocal microscope.

References

33. Yang, Y. C., Ma, Y. L., Chen, S. K., Wang C. W. & Lee, E. H. Y. Focal adhesion kinase is required, but not sufficient, for the induction of long-term potentiation in dentate gyrus neurons *in vivo*. *The Journal of neuroscience* **23**, 4072-4080, doi: https://doi.org/10.1523/JNEUROSCI.23-10-04072.2003 (2003).

34. Chao, C.C., Ma, Y. L. & Lee, E. H. Y. BDNF enhances Bcl-xL expression through protein kinase CK2-activated and NF-κB-mediated pathway in rat hippocampus. *Brain pathology* **21**, 150-162, doi: 10.1111/j.1750-3639.2010.00431.x. (2011).

35. Liu, S.Y., Ma, Y. L., Hsu, W. L., Chiou, H. Y. & Lee, E. H. Y. Protein inhibitor of activated STAT1 Ser-503 phosphorylation-mediated Elk-1 SUMOylation promotes neuronal survival in APP/PS1 mice. *British journal of pharmacology* **176**, 1793-1810, doi: 10.1111/bph.14656 (2019).

36. Tao, C. J. et al. Galectin-3 promotes Aβ oligomerization and Aβ toxicity in a mouse model of Alzheimer's disease. *Cell death differentiation*, doi:10.1038/s41418-019-0348-z (2019).
